# From gapless pan-genomes to field impact: a functional roadmap for accelerating *Brassica rapa* breeding

**DOI:** 10.1093/hr/uhag094

**Published:** 2026-03-11

**Authors:** Xu Cai, Jian Wu, Xiaowu Wang

**Affiliations:** State Key Laboratory of Vegetable Biobreeding, Institute of Vegetables and Flowers, Chinese Academy of Agricultural Sciences, Beijing 100081, China; State Key Laboratory of Vegetable Biobreeding, Institute of Vegetables and Flowers, Chinese Academy of Agricultural Sciences, Beijing 100081, China; State Key Laboratory of Vegetable Biobreeding, Institute of Vegetables and Flowers, Chinese Academy of Agricultural Sciences, Beijing 100081, China

## Introduction

*Brassica rapa* is not only a key model for polyploid genome evolution but also a major crop worldwide, with remarkable morphological diversity—encompassing leafy, root, and oilseed types. The release of the first *B. rapa* reference genome (Chiifu v1.5) in 2011 marked a transformative milestone, enabling foundational studies on genome triplication, subgenome dominance, and the genetic basis of key domestication traits, such as leaf heading and tap root formation [1, 2]. Yet for over a decade, research and breeding have been constrained by reliance on a single reference genome of Chiifu, although it was improved from the fragmented version to very continuous version [3]. Although a pan-genome based on 18 high-quality *de novo* assemblies was previously reported [4], it still relied on genomes containing numerous gaps—particularly in repetitive regions, such as centromeres and pericentromeres—leaving a substantial portion of structural and functional variation unresolved. The “reference bias” and incomplete genome assemblies obscured critical loci underlying evolution, domestication and adaptation, limiting both gene discovery and marker-assisted selection. The publication of Ma *et al*.'s [5] Science paper shatters this bottleneck with unprecedented impact. Their resource—featuring 11 T2T assemblies spanning oilseed, leafy, and root types, complemented by epigenomic, transcriptomic, and population-scale resequencing data—constitutes the most comprehensive genomic framework ever built for a vegetable crop. More than a technical triumph, it represents a paradigm shift: from viewing the genome as a static scaffold to understanding it as a dynamic, modular system shaped by repeat-driven plasticity. This new foundation not only explains how *B. rapa* diversified so rapidly after its whole-genome triplication event but also provides the precise tools needed to accelerate future improvement.

## New insights into rapid diversification

At the heart of Ma *et al*.'s breakthrough is the complete resolution of regions long deemed intractable—centromeres, pericentromeric heterochromatin, and other repeat-dense domains [5]. Using ultra-long Oxford Nanopore and HiFi reads, they assembled these regions with base-pair accuracy, revealing an astonishing level of organization within apparent chaos. Most notably, they identified five previously unknown satellite repeat families (pBrSTR92/144/197/228/497), each exhibiting striking morphotype-specific distributions. The case of pBrSTR197 is particularly illuminating: nearly absent in heading Chinese cabbage but dominating the centromere of chromosome A01 in turnip, its presence coincides with a clear shift in centromere position—a phenomenon known as neocentromere formation or centromere repositioning. This finding provides direct molecular evidence for the “modular centromere” hypothesis, wherein rapid turnover of satellite arrays enables chromosomal restructuring without disrupting kinetochore function. Such plasticity may serve as a hidden engine of reproductive isolation, allowing subpopulations to diverge genetically while maintaining fertility within groups.

Beyond centromeres, the T2T assemblies empower a systematic census of SVs—deletions, duplications, inversions, and translocations—that were previously invisible or mischaracterized. By integrating their new assemblies with earlier high-quality genomes (e.g. [4]), Ma *et al*. cataloged thousands of SVs and linked them to defining agronomic traits [4, 5]. For example, a large inversion near the *BrFLC2* locus underlies vernalization independence in tropical pak choi, enabling year-round cultivation. In heading types, tandem duplications amplify genes involved in leaf curvature and cell division, directly shaping head architecture. In turnips, a retrotransposon insertion upstream of a fleshy tap root expansion gene correlates with root swelling. Critically, these hypotheses are not left to bioinformatic speculation: the authors validate them through expression correlation across tissues, conservation across Brassicaceae, and functional knockdown via virus-induced gene silencing.

Perhaps, most transformative is the revelation that highly repetitive sequences are not evolutionary dead ends but active substrates for innovation. The T2T data show that satellite arrays can expand, contract, or relocate on evolutionary timescales short enough to impact domestication. These changes alter local chromatin states, influence 3D genome folding, and rewire gene regulatory networks over megabase distances. In this light, the ‘junk DNA’ narrative collapses. Instead, repetitive regions emerge as tunable genomic modules that buffer core functions while permitting rapid phenotypic exploration—a duality essential for both natural adaptation and human-directed selection. Future work leveraging this resource could explore how environmental stresses trigger repeat instability, or how epigenetic silencing of transposons interacts with satellite dynamics during hybridization. For breeders, the ability to track and manipulate repeat-associated SVs opens new avenues for fine-tuning complex traits that have long resisted conventional approaches.

## From genomic brilliance to breeding impact: Bridging the translation gap

Despite its brilliance, the full potential of Ma *et al*.'s resource hinges on its successful translation into applied settings [5]. Three key challenges stand in the way. First, the raw T2T sequences, even with rich multi-omics data, lack standardized, easily accessible functional annotation, making them difficult for non-specialists to use. Second, current pan-genome graphs often remain anchored to a single reference, which can bias SV discovery against sequences unique to other haplotypes, thus undermining the inclusivity of the pan-genome concept. Third, deep phenotyping across the 1720 sequenced accessions is limited to a few obvious traits; linking SVs to complex, field-relevant performance requires scalable, environment-aware phenomics.

To bridge this gap, we propose a focused, three-part strategy. We must (i) build an open, unified knowledgebase based on the 11 T2T assemblies, complete with accessible gene models, epigenetic maps, and detailed repeat annotations. This will facilitate seamless data access and empower more researchers. (ii) We should adopt multiple-reference or reference-free pan-genome frameworks that treat all haplotypes equally, ensuring an unbiased discovery of all trait-associated SVs. (iii) Finally, we need to couple pangenome-based genotyping with high-throughput field phenomics—using drones, spectral sensors, and environmental monitoring across diverse trials—to robustly connect genomic variation to real-world agronomic outcomes.

## Conclusion

With their 2026 publication of the gapless *Brassica rapa* pan-genome, Ma *et al*. [5] have delivered a very important contribution—one that enriches our perspective on plant genome evolution and domestication while serving as an invaluable resource for the entire field. Our collective responsibility now is to ensure this achievement is not left on the shelf as a scientific curiosity, but is actively used to solve real-world problems. By fostering collaborative annotation, developing equitable analytical tools, and integrating genomics with advanced field phenotyping, we can transform this exquisite map of the genome into climate-resilient, high-yielding, and nutritious cultivars within a few short breeding cycles.

## Data Availability

Not applicable as no datasets were generated or analyzed during this study.
